# Removal of Endotoxins from Bacteriophage Preparations by Extraction with Organic Solvents

**DOI:** 10.1371/journal.pone.0122672

**Published:** 2015-03-26

**Authors:** Bożena Szermer-Olearnik, Janusz Boratyński

**Affiliations:** 1 Laboratory of Biomedical Chemistry—“Neolek”, Ludwik Hirszfeld Institute of Immunology and Experimental Therapy, Polish Academy of Sciences, Wroclaw, Poland; 2 Department of Biomedical Sciences, Jan Dlugosz University, Czestochowa, Poland; University of Helsinki, FINLAND

## Abstract

Lipopolysaccharide (LPS, endotoxin, pyrogen) constitutes a very troubling contaminant of crude phage lysates produced in Gram-negative bacteria. Toxicity of LPS depends on the strong innate immunity response including the cytokines. Therefore, its removal is important for bacteriophage applications. In this paper, we present a procedure for extractive removal of endotoxin from bacteriophage preparations with water immiscible solvents (1-octanol or 1-butanol). During extraction most of the phage lytic activity is retained in the aqueous phase, while endotoxin accumulates in the organic solvent. The levels of endotoxin (expressed as endotoxin units, EU) in the aqueous bacteriophage-containing fraction determined by limulus amebocyte lysate or EndoLISA assay were exceptionally low. While the initial endotoxin levels in the crude phage lysates ranged between 10^3^ and 10^5^ EU/ml the average level after organic extraction remaining in the aqueous fraction was 5.3 EU/ml. These values when related to phage titers decreased from 10^3^-10^5^ EU/10^9^ PFU (plaque forming units) down to an average of 2.8 EU/10^9^ PFU. The purification procedure is scalable, efficient and applicable to all the bacteriophages tested: T4, HAP1 (*E*. *coli*) and F8 (*P*. *aeruginosa*).

## Introduction

During the last decades antibiotic resistance among pathogenic bacteria has increased and forms a serious threat for humans and animals. Thus, growing number of studies focuses on bacteriophages as a possible alternative for the treatment of bacterial infections [1–8]. Unfortunately, bacteriophage preparations are often contaminated by macromolecules derived from the host bacteria and culture medium. In case of phages against Gram-negative bacteria, the major pyrogen is lipopolysaccharide (LPS, endotoxin) [9]. Its presence raises one of the important concerns regarding the safety of the therapy [10–15]. The amount of endotoxins is defined by an endotoxic unit (EU) which corresponds to activity of 100 pg of *E*. *coli* lipopolysaccharide. The allowed threshold for endotoxin is established at 5 EU/kg/h for most intravenous applications [16]. However, endotoxin is much more tolerated when administered orally [7–8]. The endotoxin content of distilled water is ca. 20 EU/ml [15].

Therefore, practical processes for removal of endotoxin from bacteriophage suspensions need to be developed. Many endotoxin elimination procedures are tailored for specific bioproducts and lack generality [17]. Ultrafiltration is a common technique for purification biological macromolecules [18–19]. Affinity techniques can also be used to effectively remove LPS using immobilized ligands such as polymyxin B [20–21], l-histidine, poly-l-lysine, poly(ethyleneimine) [22], PEGylated polypeptides [23], and l-serine immobilized on polyvinylidene fiber [24]. There is a dedicated commercially available Endo-trap kit (Hyglos, Germany), which is efficient at endotoxin removal, and was also applied to bacteriophage suspensions [6, 25–26].

Our previous study on the nature of bacteriophages determined ca. –28 mV zeta potential of these nanoparticles [27] which is in agreement with Robertson et al. [28]. Such a high negative charge was an indication that ionic and ion-dipole interactions can be exploited for purification purposes. Earlier, we attempted to remove endotoxin from bacteriophage preparations using ion-exchange chromatography. In our hands, Matrex Cellulofine sulfate outperformed other more common ion-exchange media [29]. While it is difficult to assign hydrophilicity to complex molecular assemblies such as bacteriophages, our experiments with ion-exchange chromatography and electrodynamic measurements of phages suggested that extraction methods could be employed for their purification. This was particularly tempting, since preparative isolation of LPS entails a step, in which lipopolysaccharide is extracted into phenol [30] and butanol [31]. On the other hand, removal of LPS from biomolecules by extraction was previously accomplished with Triton X-114 [32–34] and tetra(ethyleneoxide) decyl ether [35]. In these methods the detergent-water system is maintained outside the miscibility area by adjusting temperature or concentration. Endotoxins partitioned favorably into organic phase, while the desired molecules remained in the aqueous phase [32–35]. A severe drawback of these methods is that the remaining residues of the detergents can interfere with the determinations of endotoxin by the Limulus Amebocyte Lysate test (LAL). For example, presence of Triton X-100 (a detergent chemically similar to Triton X-114 used for LPS removal) was shown to hamper the LAL even in trace quantities (50 ppm) [36]. Therefore, the detergent has to be removed before a reliable endotoxin determination can be performed.

In this paper, we demonstrate that endotoxin can be efficiently removed from a bacteriophage lysate by extraction with water immiscible solvents such as butanol and octanol, which are then easily removed.

## Materials and Methods

### Media

Nutrient broth (pH 7.2±0.2) consisted of 0.4 g beef extract, 5.4 g enzymatic digest of casein, 1.7 g yeast extract, 4 g peptone (BTL, Poland), and 3.5 g NaCl per liter. The components were dissolved in distilled water and sterilized in an autoclave. Sterile glucose was added to a final concentration of 1%.

### Bacterial strains

The *Escherichia coli* B strain (expressing rough LPS) was obtained from the Polish Collection of Microorganisms, IIET, Polish Academy of Sciences (PCM 1630). The strain was maintained on MacConkey agar (BTL, Poland) at 4°C.

The *Pseudomonas aeruginosa* strain was obtained from the Polish Collection of Microorganisms, IIET, Polish Academy of Sciences (PCM 2720). The strain was maintained on blood agar (BTL, Poland) at 4°C.

### Bacteriophages

The *E*. *coli* specific *b*acteriophage T4 was received from the American Type Culture Collection (Rockville, Maryland, USA). The phage strain was stored in the Polish Collection of Microorganisms, IIET, Polish Academy of Sciences.

The *E*. *coli* specific bacteriophage HAP1 (*Myoviridae*) [37] and *P*. *aeruginosa* specific bacteriophage F8 (*Myoviridae*) were obtained from the phage collection of Laboratory of Bacteriophages, IIET, Polish Academy of Sciences.

### Preparation of crude bacteriophage suspensions

Typically, bacteria culture in nutrient broth was carried at 37°C for 8–16 hours, until the optical density (OD, 565 nm) reached 1 McFarland units, which corresponded to about 10^8^ bacteria/ml [38]. At this point the culture was infected with bacteriophage 0.1 PFU / bacteria. In one case (Table 1, entries 8–9) bacteria count was estimated at 10^9^/ml (by spectrophotometry at 260 nm [38]) and T4 phage was added in 0.02 PFU / bacteria. The subsequent multiplication of bacterioiphage on bacteria cultures was carried out at 37°C for 8 h.

**Table 1:** Representative outcomes of bacteriophage purification runs with 1-octanol extraction.

Entry	Bacteriophage strain	Titer of bacteriophage (10^9^ PFU/ml)		Endotoxin level (EU/ml)^a^		Relative endotoxin content (EU/10^9^ PFU)^a^	
		Before^b^	After^c^	Before^b^	After^c^	Before^b^	After^c^
1	T4	5.0	2.0	1 950	4.0	390	2.0
2	T4	7.0	2.0	3 050	11	440	5.5
3^d^	T4	5.0	1.0	31 000	5.2	6 200	5.2
4	T4	10.0	8.0	8 700	5	870	0.6
5	T4	6.0	3.6	37 000	3	6 200	0.8
6	T4	1.9	0.9	94 000	5	49 500	5.5
7	T4	n/d^e^	3.3	100 000	8	n/d^e^	2.4
8	T4	110	50	34 000	0.9	310	0.02
9^f^	T4	110	70	34 000	50	310	0.7
10	HAP1	6.0	2.0	60 000	14	10 000	7.0
11	F8	1.9	0.9	3 800	8.0	2 000	8.9

^a^ Determined by chromogenic LAL test;

^b^ For crude bacterial lysate;

^c^ For bacterial lysate after purification but before ultrafiltration;

^d^ Preparation on a 30-liter scale;

^e^ Not determined;

^f^ Phage culture not supplemented with MgCl_2_ before extraction;

### Determination of phage titer

The titer of phages, expressed as plaque forming units (PFU), was determined using the double layer agar technique [39].

### General purification procedure

**Fig 1:**
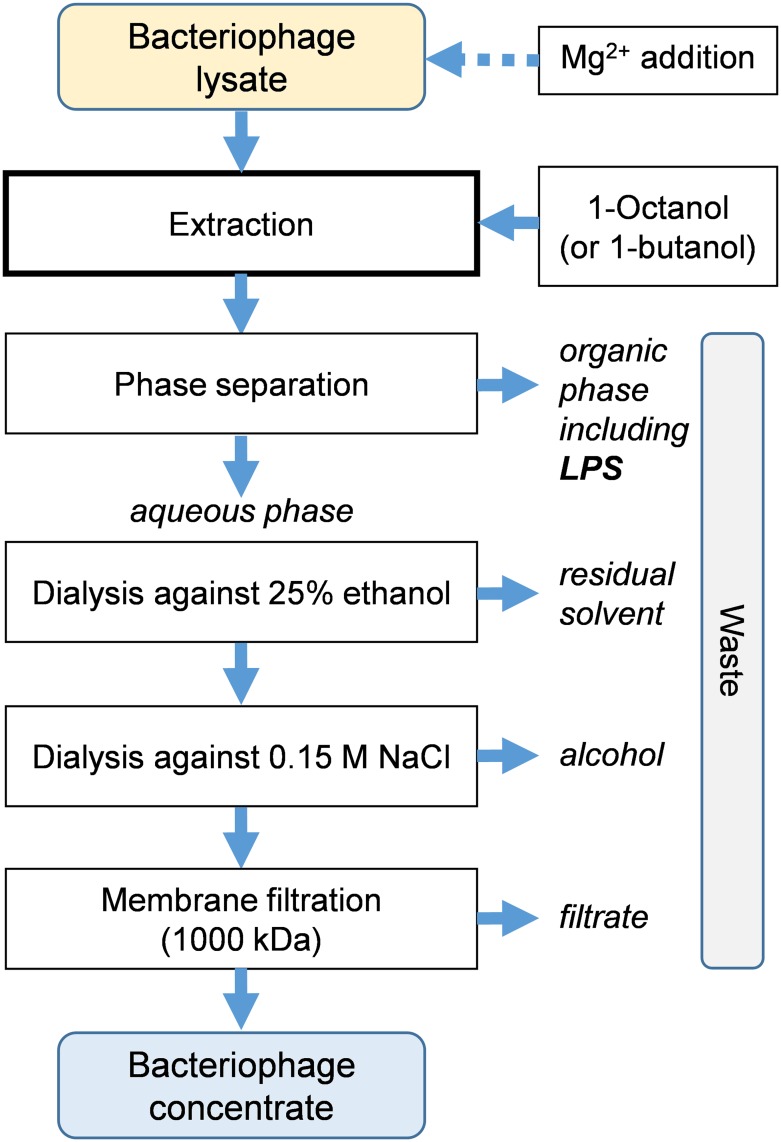
Outline of purification protocol.

Crude bacterial lysates (5 ml–20 l) were filtered through membrane filters (0.22 μm) and supplemented by adding a 0.2 M solution of MgCl_2_ to the final concentration of 0.02 M. The filtrate was incubated at 4°C for 3–24 h. The organic solvent either 1-octanol or 1-butanol (Sigma-Aldrich) was added (about 40% v/v) to the bacterial lysate and shaken for 1–3 hours at room temperature. Then, the two-phase mixture was cooled to 4°C for 1–3 hours and separated (using separatory funnel for liter-scale, or centrifugation at 4000 × g, 10 min for smaller quantities). The collected aqueous phase was dialyzed (SERVA dialysis tubing MWCO 12–14 kDa) against 25% aqueous ethanol (5 × 4 h), and subsequently against aqueous 0.15 M NaCl sterile solution (4 × 4 h). After dialysis, bacteriophage lysate was passed through a Pellicon membrane (1000 kDa, composite regenerated cellulose, EMD Millipore). Both the fraction that did not pass through the membrane (bacteriophage concentrate) and the filtrate were collected. The endotoxin levels and phage lytic activity were determined for both fractions. (Fig. 1)

### Determination of endotoxins

#### LAL test

The amount of endotoxin (as endotoxin units, EU) was determined using the end-point chromogenic Limulus Amebocyte Lysate test (LAL R-160; Charles River Laboratories International). Quantification of endotoxins was achieved using the chromogenic technique—a standard curve, consisting of three measurement points, was performed for each determination. The samples were serially diluted with apyrogenic water (supplied with the test) in 1:1 ratio until the measurement was within the range of the standard curve. For samples of crude cultures prior dilution of 1:100–1:1000 was used. Each data point was performed in triplicate. Reaction conditions were maintained according to the manufacturer’s recommendation [40].

#### EndoLISA test

Endotoxin levels of the T4 phage preparations after purification were assessed using EndoLISA (ELISA-based Endotoxin Detection Assay, Hyglos, Germany), according to the manufacturer’s instructions. Prior to analysis, the samples or standards were diluted with Binding Buffer. The samples were incubated on an assay plate overnight at room temperature with shaking. Subsequently, the plate was washed with Washing Solution and Assay Reagent was added. The fluorescent signal was detected immediately in a fluorescence microplate reader (Synergy H4, BioTek, USA) [36, 41].

In order to compensate for bacteriophage activity loss, various dilution or changes of the sample volume (eg. at the concentration step), the relative measure of endotoxin contamination is introduced, and defined as the endotoxin content per phage titer (EU/PFU).

### Particle size measurement

Particle sizes were determined by dynamic light scattering using a Zetasizer NanoZS apparatus (Malvern, UK). Measurements were performed for the final concentrate of T4 phage and the filtrate.

### The effect of ethyl alcohol on bacteriophage T4 activity

To bacteriophage T4 crude suspensions (1.2x10^8^ PFU/ml) ethyl alcohol was added to final concentration of 10, 20, 25, 30, 40, 60% v/v. The mixture was then incubated at ambient temperature (18–22°C). After 24 h of incubation the samples were diluted 1000-fold with aqueous 0.15 M NaCl, and phage titer was assayed.

### Statistical analysis

Confidence intervals at the 95% probability level (CI95%) were calculated assuming normal distribution. They are defined as ±1.96×*SE*, where *SE* is a standard error of the mean.

## Results and Discussion

### Purification procedure

In this work we investigated the partitioning of bacterial phage lysates in a biphasic liquid system. To facilitate the downstream processing we decided to use easily removable, water-immiscible organic solvents for extraction, namely 1-octanol and 1-butanol. The purification procedure begun with the removal of debris from the T4 bacteriophage lysate of *E*. *coli* by filtration, and for smaller scales prior centrifugation (8000×g, 30 min). Then the filtered suspension was mixed with the selected higher alcohol and extracted. The bacteriophages remained in the aqueous phase (Fig. 1, Table 1), whereas the endotoxins were transferred into the organic phase. Interestingly, after evaporation of 1-butanol fraction (rotary evaporator and evacuation on a freeze-dryer) and dissolution in the corresponding volume of aqueous NaCl, the total endotoxin activity detected by the LAL test (1.9–3.4×10^7^ EU/ml) exceeded that of the initial lysate (up to 10^5^ EU/ml) by two orders of magnitude. No significant accumulation of endotoxins was found at the interface. The partitioning of lipopolysaccharides into butanol from bacteria suspension was previously used by Morrison and Leive for isolation purposes [31]. In our hands, both 1-octanol and 1-butanol provided similar results in terms of endotoxin removal efficiency and phage titre preservation, also other water-immiscible liquids such as dichloromethane or rapeseed oil were effective. Nevertheless, we found 1-octanol to be most practical also due to easier phase separation.

Determination of endotoxin level directly after extraction was impossible, while we found that residual 1-octanol in the aqueous phase disabled the LAL test. False “0 EU/ml” value was obtained for unpurified bacteriophage samples saturated with the extraction solvent. The solubility of 1-butanol and 1-octanol in pure water is 80 g/l and 0.5 g/l, respectively. Thus, sequential dialyses against 25% ethyl alcohol and 0.15 M aqueous NaCl were employed. The purpose of the first was to remove organic solvents (<10 ppm as determined by gas chromatography), while the second dialysis removed ethyl alcohol. In a separate experiment, we have verified that T4 bacteriophages retained at least 90% of their antibacterial activity following 24 h storage in up to 30% ethyl alcohol. Significant loss of activity of T4 was observed at higher concentrations: 0.2% of initial activity was retained after storage in 40% ethanol, while in 60% ethyl alcohol the bacteriophage activity was very low, but still detectable.

The last step of the procedure, membrane filtration (1000 kDa cut-off) was aimed to remove macromolecules originating from the culture medium and bacteria. The filtration also concentrated the bacteriophages but did not contribute to the removal of residual endotoxins at this point. Only trace lytic activity passed to filtrate (<0.02%). Light scattering measurements in the bacteriophage concentrate exhibited a prominent signal at 130±18 nm of low polydispersity. According to Robertson et al. this signal is attributable to T4 bacteriophage nanoparticles [28]. On the other hand, measurements in the filtrate revealed broad range of particle sizes (0.7 to 650 nm) and high polydispersity index (PDI: 0.737). The apparent larger hydrodynamic radii of some particles found in the filtrate than in the concentrate is probably a result of aggregation.

### Scope and modifications

The proposed method for endotoxin removal from crude suspension was developed on the example of bacteriophage T4 (Fig. 1, Table 1). The extraction procedure efficiency was assessed by simultaneous analysis of endotoxin levels coupled with control of phage titers. Results of 8 representative experiments are summarized in Table 1, entries 1–8. The procedure provided bacteriophage preparations with low endotoxin activity (average 5.3 EU/ml; CI95% 3.3–7.3 EU/ml). This corresponds to decreases by two to four orders of magnitude in the endotoxin activity in pure preparations compared to crude suspension, as determined by chromogenic LAL test. On the other hand, the extraction procedure did not cause much deterioration of phage titers. Typically half of the initial phage lytic activity was retained after purification process (CI95% for recovery rate, 32–60%). Thus, the contamination level, defined as the endotoxin content per phage titer (EU/PFU), decreased by more than 99%. The contamination of the final product was on average 2.8 EU/10^9^ PFU, and CI95% was in 1.3–4.3 EU/10^9^ PFU range.

In comparison, our method applying Cellulofine sulfate gave preparations nearly 10 times more contaminated (20 EU/10^9^ PFU) and in poor yield [29]. On the other hand, newly introduced EndtoTrap was used for bacteriophage preparation [6, 25–26], however the cost of the process is prohibitive. Morello et al. used EndoTrap blue column for endotoxin removal from the P3-CHA and PAK-P3 bacteriophages against *P*. *aeruginosa*. The resultant bacteriophage preparation contained down to 0.1 EU/10^9^ PFU [24]. However, Maillard et al. achieved only marginal purification of bacteriophage cocktail against *P*. *aeruginosa* [26].

The bacteria and phage content in the process of making lysate is subject to variations due to multiple factors. Some of them include volume of the culture, initial bacteria concentrations, conditions of growing bacteria, initial state of the culture and added phage quantity. All of these translated to the unpredictability of the composition of the broth used for purification. The variations in both the bacteriophage counts (range 1.9×10^9^–1.1×10^11^ PFU/ml; Table 1, entries 6 and 8) and endotoxin levels (range 1.9×10^3^–1.0×10^5^ EU/ml; Table 1, entries 1 and 7) spanned independently for two orders of magnitude. However, they did not influence the outcome of the procedure, and the endotoxin level in the final product was in much narrower range (0.9–11 EU/ml), and no correlation was found between the relative contamination of the raw lysate and that of the final product (R^2^ = 0.49).

The versatility of the bacteriophage lysate extraction with organic solvents was tested using two other bacteriophages, the *E*. *coli*-specific phage HAP1 and the *P*. *aeruginosa*-specific phage F8 (all myoviruses). The organic extraction with 1-octanol was performed as described for T4 and the results are shown in Table 1. The organic extraction decreased the titers of the HAP1 and F8 lysates to 47 and 33% of the original titers, respectively. However, the endotoxin levels of the lysates dropped from 6×10^4^ and 3800 to 14 and 8 EU/ml, respectively. The latter values corresponded to 7.0 and 8.9 EU/10^9^PFU.

Bivalent ions were shown to be beneficial for stabilization of bacteriophage suspension and preservation of lytic activity [42]. Therefore, we supplemented the broth prior to extraction with magnesium chloride (20 mM). Omission of MgCl_2_ resulted in less efficient extraction of endotoxin as demonstrated by the results of two parallel purification runs starting from the same raw lysate with and without added MgCl_2_ (Table 1, entries 8–9). Magnesium and other divalent ions were shown to affect chain mobility, aggregate structure and biological properties of LPS from *Salmonella minnesota* [43]. It was also proven that these ions influence bacteria/lambda phage adsorption [44], and lytic activity [45]. Although we cannot provide a direct explanation for the role of magnesium ions in our procedure the results were encouraging for their use.

### Validity

The reliability of the LAL assay showing low endotoxin activity in the preparations was verified. For two different samples of freshly purified T4 bacteriophage, an alternative assay EndoLISA was performed in addition to the LAL. The LAL test gave 5.1 and 10.4 EU/ml results, while EndoLISA showed 0.8 and 1.4 EU/ml, respectively. Although these values are numerically different, they are qualitatively consistent with low endotoxin content in the final preparation. The 7-fold difference between the tests can possibly arise from different mode of test operation (*Lymulus* lymph fluid vs. bacteriophage protein) and thus different selectivity of the two assays. Both are also susceptible to different sets of pertubents [36]. The two assays were also compared after addition of test-specific endotoxin standards (0.6–6 and 2.5–250 EU/ml for LAL and EndoLISA, respectively) to the measured bacteriophage sample. Both tests showed good response to the added endotoxin, R^2^ was 0.9 and 0.99 for the LAL and EndoLISA tests, respectively. LAL test is the current reference assay, however in the course of our experimentation we have found the test to provide some irreproducible results on few isolated occasions. As mentioned previously with the LAL test, the total activity of endotoxin obtained from the organic phase not containing bacteriophages accounted for 10^7^ EU/ml, that is far beyond the activity assayed in the initial broth (10^5^ EU/ml).

## Conclusions

In this work we have demonstrated that organic solvents such as 1-butanol and 1-octanol can be used to remove efficiently endotoxin from aqueous bacteriophage lysates at the same time retaining the infectivity of the bacteriophages. The presented method is efficient, scalable and cost-effective and therefore a significant alternative for endotoxin removal from bacteriophage lysates.
